# The role of forests in the EU climate policy: are we on the right track?

**DOI:** 10.1186/s13021-023-00234-0

**Published:** 2023-07-30

**Authors:** Anu Korosuo, Roberto Pilli, Raúl Abad Viñas, Viorel N. B. Blujdea, Rene R. Colditz, Giulia Fiorese, Simone Rossi, Matteo Vizzarri, Giacomo Grassi

**Affiliations:** 1 Joint Research Centre, European Commission, Ispra, Italy; 2 Independent researcher providing service to the Joint Research Centre, European Commission, Ispra, Italy; 3 Present Address: Basque Centre for Climate Change (BC3), Scientific Campus of the University of the Basque Country, Sede Building, 1, 1st floor, 48940 Leioa, Spain; 4 Present Address: Università degli Studi di Milano, Milano, Italy

**Keywords:** Carbon sinks, Climate legislation, European Union, Forest management, Forest monitoring, LULUCF

## Abstract

**Background:**

The European Union (EU) has committed to achieve climate neutrality by 2050. This requires a rapid reduction of greenhouse gas (GHG) emissions and ensuring that any remaining emissions are balanced through CO_2_ removals. Forests play a crucial role in this plan: they are currently the main option for removing CO_2_ from the atmosphere and additionally, wood use can store carbon durably and help reduce fossil emissions. To stop and reverse the decline of the forest carbon sink, the EU has recently revised the regulation on land use, land-use change and forestry (LULUCF), and set a target of − 310 Mt CO_2_e net removals for the LULUCF sector in 2030.

**Results:**

In this study, we clarify the role of common concepts in forest management – net annual increment, harvest and mortality – in determining the forest sink. We then evaluate to what extent the forest sink is on track to meet the climate goals of the EU. For this assessment we use data from the latest national GHG inventories and a forest model (Carbon Budget Model). Our findings indicate that on the EU level, the recent decrease in increment and the increase in harvest and mortality are causing a rapid drop in the forest sink. Furthermore, continuing the past forest management practices is projected to further decrease the sink. Finally, we discuss options for enhancing the sinks through forest management while taking into account adaptation and resilience.

**Conclusions:**

Our findings show that the EU forest sink is quickly developing away from the EU climate targets. Stopping and reversing this trend requires rapid implementation of climate-smart forest management, with improved and more timely monitoring of GHG fluxes. This enhancement is crucial for tracking progress towards the EU’s climate targets, where the role of forests has become – and is expected to remain – more prominent than ever before.

## Background

### Forests in the EU climate policy

The EU climate law [1] sets the objective of EU climate neutrality by 2050, and many EU member states have set similar or more ambitious national targets. To meet these goals, phasing out nearly all greenhouse gas (GHG) emissions is necessary, and any remaining emissions need to be counterbalanced through carbon dioxide (CO_2_) removal. This is expected to be achieved through an increase of CO_2_ removals in the land use, land-use change and forestry sector (LULUCF), and over time also through technological solutions, such as by carbon capture, use and storage. In this context, forests play a particularly crucial role, as they are currently the main option at hand to remove CO_2_ from the atmosphere. In addition, wood use may further contribute by storing carbon durably and help reducing emissions from energy-intensive materials and fossil energy [2]. As a steppingstone towards climate neutrality, the EU has agreed on a binding target of reducing its overall net emissions by 55% by the year 2030, compared to the total net emissions in 1990 [1]. To achieve this target, the Commission proposed to revise all related legislation, including the legislation on LULUCF [3].

The first legally binding commitments for the EU LULUCF sector were set by Regulation 2018/841 (hereafter ‘2018 LULUCF regulation’) for the years 2021–2025 [4]. Under this regulation, each EU member state must ensure that accounted emissions from LULUCF are entirely compensated by an equivalent accounted removal of CO_2_ from the atmosphere through action in the sector; this is the so called “no debit” rule. Translated to reported net removals in the scope of the regulation, the EU accounting benchmark for LULUCF is approximately − 229 MtCO_2_e/y for the period 2021–2025 (the negative sign denotes a net removal of CO_2_e from the atmosphere, i.e. a sink; a positive sign would denote a net emission to the atmosphere) [5]. This commitment is based on accounting rules that compare the net emissions and removals during 2021–2025 with the period 2005–2009 for croplands and grasslands and with the projected member state-specific forest reference levels (with a reference period of 2000–2009) for forest land and harvested wood products. The 2018 LULUCF regulation is widely seen as a step forward in credibility and ambition compared to the commitments under the Kyoto Protocol [6–9]. However, the accounting rules, especially the forest reference levels, were found to be too complex to implement and sensitive to the applied methodological assumptions [5, 10–13], and have consequently faced critique on the legal legitimacy of such a technically complex approach [14].

In addition, an increasing concern is that in recent years the EU LULUCF sink has developed counter to the climate objectives and is now showing a clear decreasing trend. To stop and reverse this trend, and to be consistent with the EU climate law, the 2018 LULUCF regulation has been revised [15] (hereafter ‘revised LULUCF regulation’), notably for the requirements from 2026 onwards. The revised LULUCF regulation simplifies the way the sector’s climate contribution is considered toward the overall climate objectives, and sets a target of -310 Mt CO_2_e to be reached by 2030 in the EU, including all reported LULUCF categories. Furthermore, the revised LULUCF regulation introduces requirements on improved methodologies and more detailed monitoring, and is complemented by other new policy instruments, such as proposals for a Nature Restoration Law [16], Sustainable Carbon Cycles [17] and Carbon Removal Certification [18], the new EU forest strategy for 2030 [19], and upcoming proposals for a new Soil Health Law [20, 21] and for a new framework for forest monitoring and strategic plans [22]. Together, these policies aim at integrating the forest sink in the broader ecological and economic context and promoting business opportunities for enhancing the LULUCF sink.

Similarly to the 2018 LULUCF regulation, the revised LULUCF regulation ensures that biomass use for energy is accounted for in the overall EU climate policy. To avoid double-counting and in line with international agreements and practice [23, 24], CO_2_ emissions from biomass combustion are rated zero in the energy sector, as they are already considered in the LULUCF sector as a loss of carbon at the time when the biomass was harvested. This connection between the energy and LULUCF sectors is a crucial prerequisite for including biomass as a part of EU’s and its member states’ targets for renewable energy under Renewable Energy Directive [25], currently under revision). As a result, the EU member states need to consider the trade-offs between promoting biomass as a renewable energy source, and its implications in the LULUCF sector [26].

With the aim of ensuring strong emissions reduction in other sectors, the overall contribution of the LULUCF sector to EU’s economy-wide target of − 55% by 2030 is limited to a maximum of − 225 Mt CO_2_e/y [5]. Achieving the LULUCF sector target of -310 Mt CO_2_e in 2030 will elevate the total EU GHG emission reduction to approximately 57% compared to 1990 [27].

### Reporting of the greenhouse gas emissions and removals in forests

**Fig. 1:**
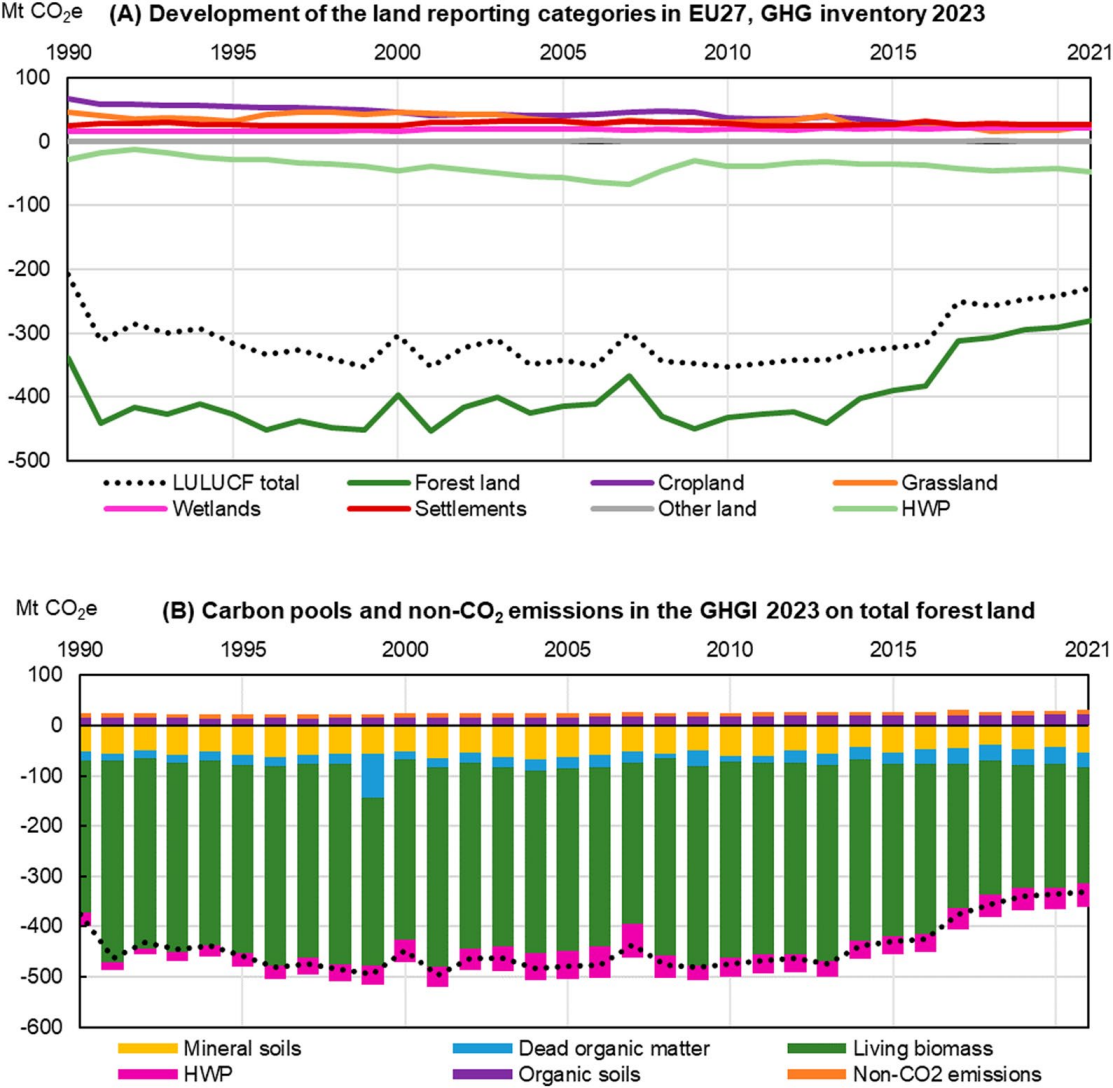
Time series 1990–2021 of net emissions and removals in LULUCF land use categories (**A**) and in the different forest carbon pools and subcategories, including harvested wood products (HWP) (**B**), as reported in the EU GHGI 2023 [28]. Estimates in this study reflect the geographical scope of the EU and the global warming potential of the fifth Assessment Report of the IPCC, as agreed for the EU climate target in 2030

Within the national GHG inventories (GHGI), the LULUCF sector encompasses the categories forest land, cropland, grassland, wetlands, settlements and other land, and also includes carbon stock changes from harvested wood products. The EU GHGI, as a sum of the members states’ GHGI, reports a total LULUCF sink of at least − 300 Mt CO_2_e/y for 1995–2016, with a clear decrease thereafter to − 230 Mt CO_2_e in 2021 [28]. The net LULUCF sink removed an equivalent of ca. 7% of the EU’s GHG emissions (excluding LULUCF) in 2021. The most important category in the LULUCF sector is forest land (Fig. 1a), with net removals of − 281 Mt CO_2_e at EU level in 2021. This net sink in forest land is a combination of ‘forest land remaining forest land’ (land considered as forest for more than 20 (in some cases 30) years, − 245 Mt CO_2_e in 2021), ‘land converted to forest land’ in the last 20 (or 30) years (− 41 Mt CO_2_e in 2021) and emissions from drainage and rewetting (+ 6 Mt CO_2_e in 2021, which are reported on the level of total forest land). In addition, the increase in carbon stock in harvested wood products (HWP, including sawn wood, wood panels, and pulp and paper produced from domestic biomass) corresponded to reduction of emissions of − 47 Mt CO_2_ in 2021.

As shown in Fig. 1b, the most important component of the reported forest sink – and also of the net LULUCF sink – is the annual accumulation of carbon in living biomass, which includes all living parts of trees: stems, stumps, roots, branches, bark, seeds, and foliage. Therefore, changes in living biomass are largely driving the changes observed in the forest sink, and even in the whole LULUCF sector, as is clearly shown in the decline of the sink reported after 2013. Given the importance of changes in living biomass in determining the whole forest sink, we hereafter use the term “forest sink” to refer to changes in living biomass in forest land, unless stated differently.

### Aim of the study

This study has three main objectives. The first is to clarify the role of common concepts in forest management – net annual increment and harvest, including the impact of mortality – in determining the forest sink. The second objective is to evaluate to what extent the forest sink, including HWP, is on track to meet the climate goals of the EU. For this assessment we use data from the latest national GHGIs [28] in comparison to the assessment underlying the revised LULUCF regulation [5]. By using a forest model (Carbon Budget Model, CBM), we also analyse the development of the future net annual increment, harvest, and forest sink in the EU, assuming that current forest management practices continue. The third objective is to discuss potential strategies for improving forest sinks, taking into account adaptation and resilience that are essential for ensuring sustained climate action.

## Results and discussion

### How forest increment and harvest impact the carbon stock and sink

The indicators of climate policy and forest management are not always directly comparable due to a difference in (1) scope, (2) units and (3) goals. First, climate-related indicators consider all tree parts and fluxes between carbon pools and the atmosphere, while forest data typically refers to only the merchantable wood (usually tree stem, sometimes also tree tops and branches), i.e. mostly reflecting above-ground living biomass carbon pool. Second, the climate commitments are expressed in terms of carbon dioxide or its equivalent (CO_2_, CO_2_e when other GHG are included), but forest data such as growing stock, increment and harvest are typically expressed in cubic meters of volume. Third, one of the objectives for sustainable forest management is typically sustainable yield: the possibility to sustain similar harvest volumes also in the future (with considerations to forest vitality and biodiversity). Over large areas and over time, this is typically understood to mean that harvest does not exceed the net annual increment, so that the growing stock is maintained or increased over time. For the EU climate policy, however, the important concept is maintaining and enhancing the sinks. It is therefore essential that the relationship between the indicators of forest management and those of climate policy are well understood.

“Sink” is a term used for a process that removes a greenhouse gas from the atmosphere, while a “source” is a process that releases greenhouse gases into the atmosphere. Living trees act as both: photosynthesis absorbs CO_2_, while respiration releases CO_2_. Tree growth shows that more CO_2_ is absorbed than released, making the tree a net sink. The same concept is valid also in the larger scale: if the total growing stock of the national forests increases, the forests are a net sink; if growing stock decreases, the forests are a net source. The more the stock increases (or decreases), the stronger is the net sink (or net source) – and if the stock remains at a constant level, the net sink is zero, i.e. there is steady state between trees and the atmosphere. In other words, the sink in living biomass of forests is the first derivative of the growing stock.

**Fig. 2:**
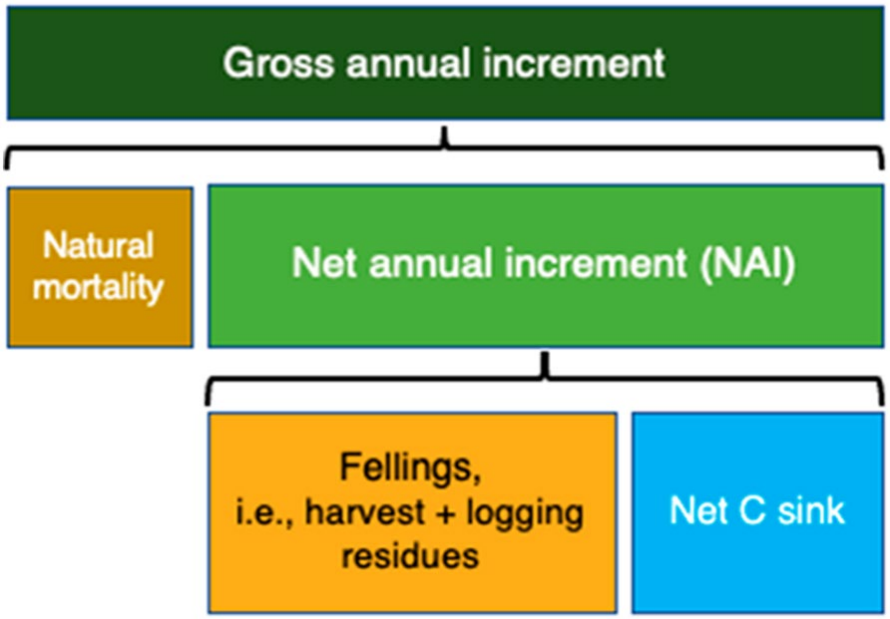
Conceptual illustration of the main components determining the net carbon sink in living biomass in forests, per area unit. Green colours indicate biomass growth; brown colours indicate biomass loss from the forest; and blue indicates the net sink of carbon in the forest. Natural disturbances are here included partly in natural mortality and partly under fellings (salvage logging). Adapted from [29]

In forest management terms, the net forest sink (in living biomass) in a given area depends on the relationship between gross annual increment, natural mortality and fellings (i.e., harvest plus logging residues left on site), as illustrated in Fig. 2. The net forest sink can increase if the net annual increment (NAI; gross annual increment minus the natural mortality) increases, if the natural mortality decreases, if the fellings decrease, or a combination thereof. Natural disturbance events (wildfires, windthrow, insect outbreaks, etc.) usually affect all these elements, because typically a natural disturbance causes more natural mortality, more harvest (salvage logging to remove damaged trees, or sanitary fellings to prevent the spread of the damage), and often also decreases the NAI. On the national level, the net forest sink is also dependent on the total forest area and its changes.

**Fig. 3:**
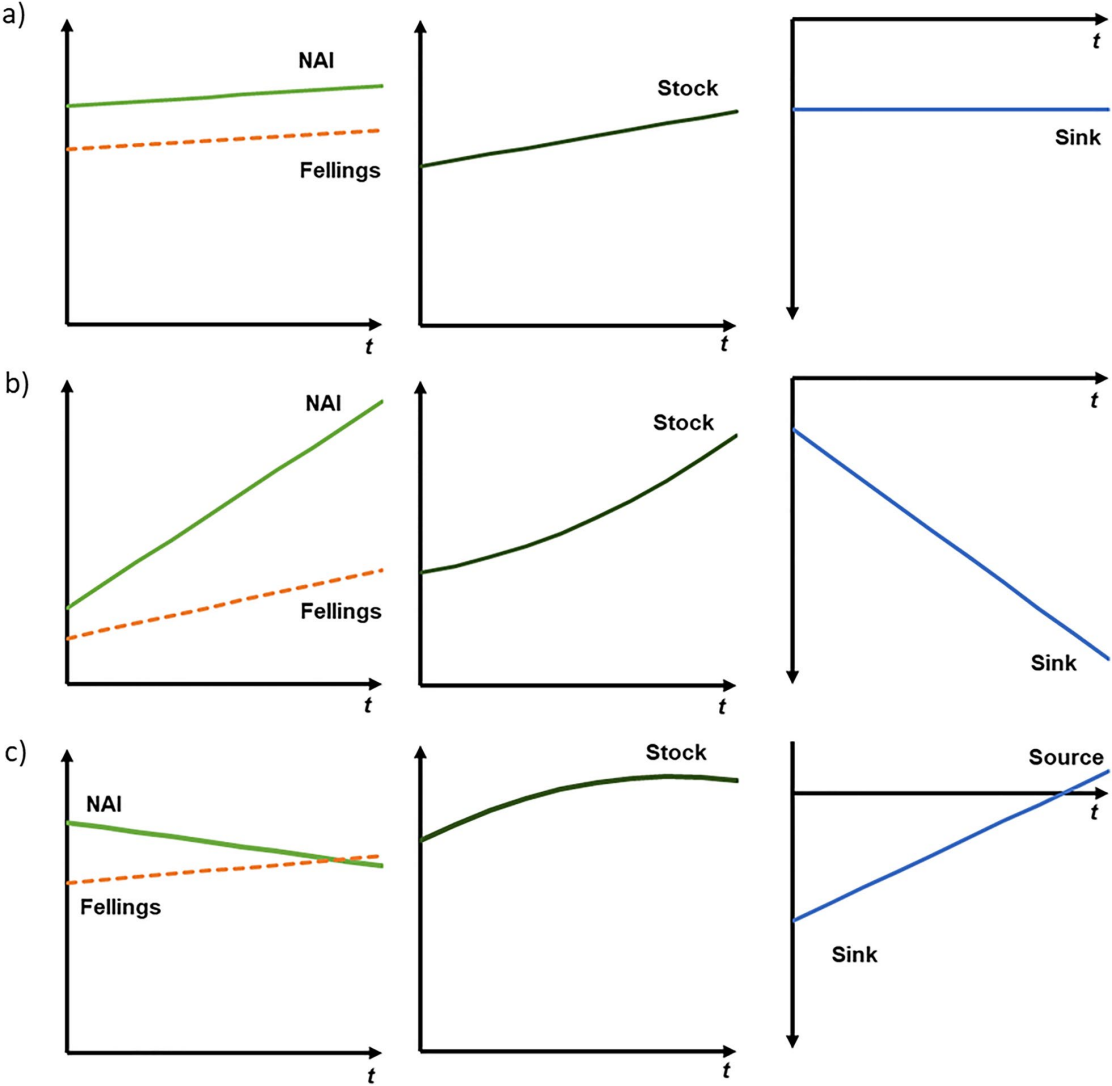
Conceptual illustration of the relationship between net annual increment (NAI), fellings, forest growing stock, sink and source in living trees over time. Other tree mortality is not considered in this figure. Note that, in the right column, net sink is shown below the x axis because it is conventionally denoted with a negative number in GHGI, while net source is denoted as a positive number. **a** NAI and harvest are stable (or increase at the same rate)—if NAI is higher than harvest, stock increases linearly and the sink is stable; **b** NAI increases more than harvest (or NAI remains constant and harvest decreases)—the stock increases exponentially and the sink increases linearly; **c** difference between NAI and harvest gets smaller—the stock increases at a slowing rate and the sink decreases. Sink turns into a source when harvest exceeds NAI; this is also when the stock starts to decrease

Figure 3 illustrates several possible relationships between NAI, fellings, carbon stock, and carbon sink or source. It is important to note that pure maintaining the sink in the forest means that the forest stock is continuously increasing, i.e. that NAI remains higher than harvest. In contrast, increasing the sink requires that NAI increases at a faster rate than the harvest, or inversely that harvests decrease relative to NAI. That is, enhancing the sinks requires a substantially stronger effort from forest management than the traditional concept of sustainable yield, which is achieved when the harvest remains below increment.

### Development of the EU forest sink to date

The overall forest growing stock has almost continuously increased since the 1950s in the EU [30, 31], which is reflected in a sustained forest sink. Further, the rate of the growing stock increase was enhanced over time, i.e. NAI was growing faster than fellings and this resulted in a progressively stronger sink (as in Fig. 3b). The annual net sink in European forests has been estimated to have increased by almost five-fold from 1950 to 1999 [30]. This trend is explained by a number of factors: environmental factors such as CO_2_ fertilization and temperature increase favoured the growth of the forests (e.g., [32, 33]; forests were regenerated and replanted in the first half of the 20th century, or immediately after the Second World War, after a long period of over-exploitation, which resulted in an overall younger age structure than today [34]; and changed forest management, including favouring planted forests and wood production-oriented management with faster growing species monocultures, even-aged stands, and plant breeding [34–37].

However, the historically increasing forest sink was reported to stabilize about a decade ago [31], due to NAI and harvest becoming approximately stable (similarly to Fig. 3a). Today, the EU and many member states’ inventories show that the sink has turned towards a clear decline in many countries, leading to an overall decrease of the forest sink in the EU (as the Fig. 3c). Countries’ reporting to the UNFCCC [38] and literature analysis [39–42] suggest that there are four main drivers of the forest sink decline in the EU: decreased afforestation (‘land converted to forest’, which includes natural forest expansion), decreased gross increment, increased natural mortality (including both annual natural losses and natural disturbances) and increased harvest.

**Fig. 4:**
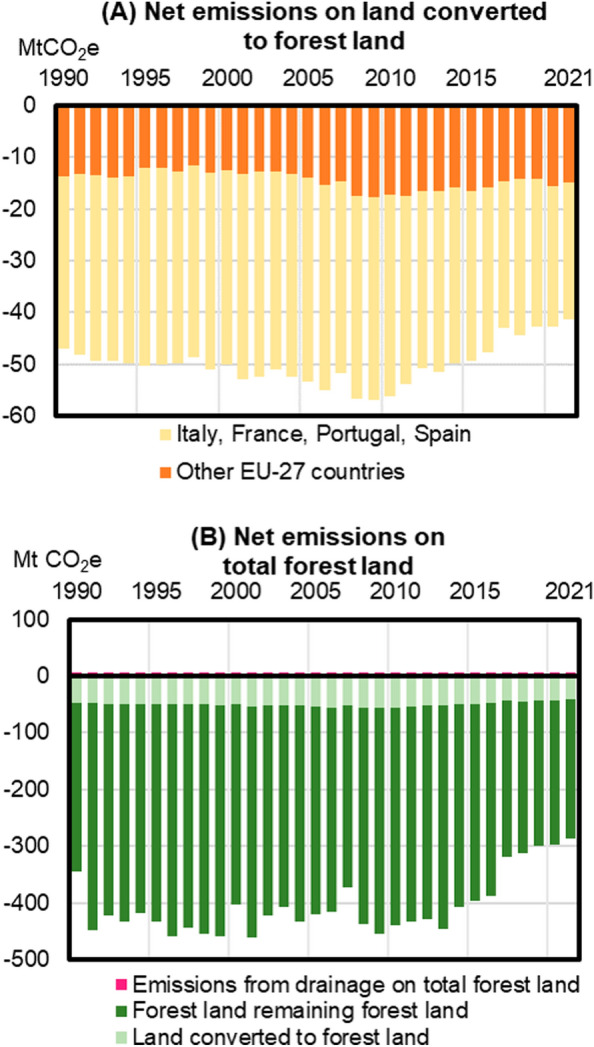
Net removals from land converted to forest land **A**, (i.e., afforestation) and from total forest land **B** in the EU-27 over time, including all carbon pools and greenhouse gases as reported in the GHGI 2023 [28]. Emissions from drainage in **B **are from Common Reporting Format table 4(II)

According to the EU GHGI, the sink resulting from afforestation followed an increasing trend from 1990 to 2008, where after it has declined by approximately 15 MtCO_2_e/y by 2021 (Fig. 4a). This decline was driven mostly by the trends observed in France, Spain, Portugal and Italy, and corresponds to ca. 10% of the decline of the total forest land sink from 2008 onwards (Fig. 4b). By comparison, emissions from deforestation slightly decreased in the same period, from ca. +35 MtCO_2_e/y in 2008 to + 28 MtCO_2_e/y in 2021.

To investigate the reasons for the larger remaining part of the rapidly decreasing forest sink, it is necessary to look at the clearly linked development of net increment, natural mortality and harvest levels in the existing forests (reported as forest land remaining forest land). Several countries report on adverse weather conditions or natural disturbances in the recent years, such as severe droughts (Czechia, Austria, Germany, Poland, Slovakia, France, Sweden), exceptional wildfires (Portugal, Italy), windstorms (Italy, Austria), and abrupt freeze (Slovenia) in their National Inventory Reports [38]. In central Europe, these events have been followed by an exceptionally strong attack of bark beetles, leading to unprecedented salvage logging (i.e., increased harvest) of the damaged or threatened trees [39]. An increase in natural mortality is also reported (e.g., [40], for France). This trend is expected to further develop in the coming decades due to climate change [41, 42]. Finland and Sweden, where the net increment was observed to increase steadily over decades, now report a stabilizing or even decreasing net increment rate, whereas harvest level has continued to increase in both countries. Many East European countries (Bulgaria, Estonia, Latvia, Croatia) have recently started to incentivize forest management through increased harvesting, after many years of limited management which led to a skewed age structure of relatively old forests. All these factors combined point to an overall likely decrease of the net increment rate on the EU-level, although up-to-date data is not available from all member states [43]. At the same time, the harvest levels have continued to increase.

### Is the current forest sink on the right track?

As mentioned before, the revised LULUCF regulation [15] sets an EU target of − 310 Mt CO_2_e in the year 2030 for the whole LULUCF sector. The Commission’s impact assessment [5] for the legal proposal demonstrated that additional mitigation efforts beyond the modelled scenario will be needed. According to the underlying modelling, cost-efficient solutions will require small to moderate additional investments to enhance the forest sink and reduce the emissions from other land use categories. As a basis for the target, the average of net removals of 2016–2018 from the GHGI 2020 (− 268 Mt CO_2_e/y) were used. Reaching the 2030 target of − 310 Mt CO_2_e would therefore require an increase of annual net removals by 42 Mt CO_2_e by 2030. As the contribution of forests and other land use categories in the modelling was roughly equal, it can be understood that in 2030 ca. 21 Mt CO_2_e of the additional net removals would come from forests.

**Fig. 5:**
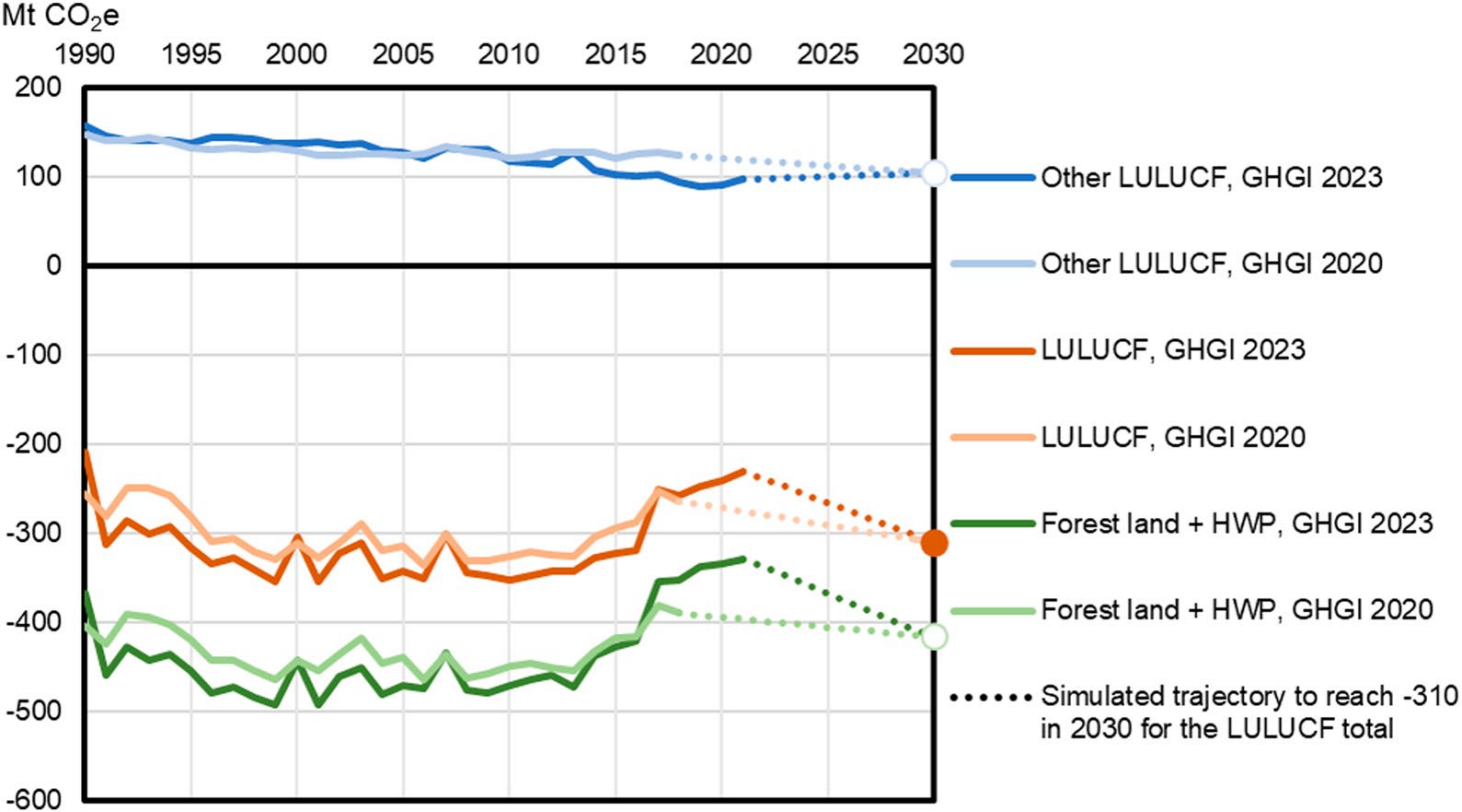
Trends of EU-27 net emissions and removals for LULUCF, Forest land + HWP, and other LULUCF categories, as reported in the GHGI 2020 and GHGI 2023, and a comparison between the trajectories needed to reach the agreed target of − 310 Mt CO2e for the total LULUCF sector in 2030. The additional net removals initially needed for the − 310-target are assumed to be split equally between Forest land + HWP and other LULUCF categories, reflecting the modelling underpinning the Impact Assessment for the revised LULUCF regulation (EC 2021). The figure reflects the geographic scope of the EU-27 (as in the LULUCF regulation), excluding non-EU territories of France and Denmark

Table 1; Fig. 5 illustrate how the trend and the absolute level of LULUCF emissions and removals have evolved after the legislative LULUCF proposal was made in 2021. There are two components to the changes: development over time, which is here analysed based on the latest available data (GHGI 2023) [28]; and recalculations to the past reporting, on which we compare the latest data (GHGI 2023) to the inventory reporting used at the time of the legislative proposal (GHGI 2020, see [44]).

In terms of trend, the latest EU GHGI [28] shows that in 2021, the total reported LULUCF net sink is 46 Mt CO_2_/y weaker than the average of 2016–2018. All those net losses occurred in forest land and in HWP, whose combined net sink decreased by 48 Mt CO_2_e between the period 2016–2018 and 2021; in contrast, other LULUCF categories decreased their aggregated net emissions by 2 Mt CO_2_e/y over the same time.

In addition, the historical estimates have been revised. For the period 2016–2018, GHGI 2023 reports an annual net sink in forest and HWP that is 18 Mt CO_2_e weaker than the reporting for that period in GHGI 2020, while the net sink for the entire LULUCF sector was recalculated to be 8 MtCO_2_e stronger. The revisions are due to more complete reporting of different carbon pools, which explains especially the recalculation of other LULUCF categories, as well as changes in methodology that are reflected over the whole time series. In forest land, however, an important reason for the recalculations of the most recent years is the time lag in the inventories. Forest estimates in the GHGI are based on national forest inventories, which are typically carried over several years, and the annual GHGI estimates for the latest years are recalculated as more data becomes available.

Overall this means that, in the light of the 2023 inventory, the EU LULUCF sector target is drifting further away: on the EU-level, instead of 42 MtCO_2_e/y as calculated as a basis for the revised LULUCF regulation, the sector now needs to increase its annual sink by 80 MtCO_2_e/y from the 2021 level to reach the agreed 2030 target of − 310 MtCO_2_e/y. This development indicates that, while the net emissions in non-forest categories are slowly going in the right direction, i.e., reducing emissions, the sink in forest and HWP is rapidly going in the opposite direction (Fig. 5).

It should be noted that GHG reporting is a continuous process, and recalculations will occur also in the subsequent inventories. These recalculations will reflect new and better data and methods, including the improvements expected under the revised LULUCF regulation, the UNFCCC review process, and the comprehensive EU review of the member states’ GHGIs that will take place in 2025. The final compliance check under the revised LULUCF regulation will be made against the 2032 GHGI submission [15].

**Table 1:** Comparison of the EU-27 reporting in GHG inventories 2023 and 2020

Comparison of the GHGI 2020 reporting used as a basis for revised LULUCF regulation and the most recent GHGI 2023					
All units are in Mt CO_2_e.	GHGI 2020	GHGI 2023		Change	
	Average reported emissions 2016–2018 (A)	Average reported emissions 2016–2018 (B)	Reported year 2021 (C)	Between reported emissions 2016–2018 and 2021 in GHGI 2023 (B vs. C)	Between GHGI 2020 and GHGI 2023 for reported emissions 2016–2018 (A vs. B)
LULUCF total	− 268	− 276	− 230	+ 46	− 8
Forest land + HWP	− 395	− 377	− 329	+ 48	+ 18
Other LULUCF	+ 126	+ 100	+ 98	− 2	− 26

In the reported change, the numbers refer to net emissions; change indicated with a positive number is therefore an increase in net emissions (or decrease of sink), while negative number denotes a decrease in net emissions (or increase of sink). The table reflects the geographic scope of the EU-27 (as in the LULUCF regulation), excluding non-EU territories of France and Denmark

### Projected development of the forest increment, harvest and sink in the EU

To analyse the prospects for the future forest sink, we project the development of NAI, harvests and forest biomass sinks until 2050 using the forest Carbon Budget Model (CBM, [45]). In this analysis, the forest area was kept constant over time at 158 Mha, reflecting the total forest area in year 2016. From 2016 onwards, forest management was assumed to continue the forest management practices that took place in the period 2000–2015, like in [46]. A similar logic was used by the EU member states when projecting the forest reference levels for the years 2021–2025 under the 2018 LULUCF regulation, but for the reference period 2000–2009 [9, 13]. It is important to note that this is not a policy scenario, but instead a simulated continuation of business-as-usual management aimed to explore the evolution of key variables. Furthermore, potential future negative impacts of climate change (e.g., a reduction of NAI, or an increase of natural disturbances) are not included in this analysis.

**Fig. 6:**
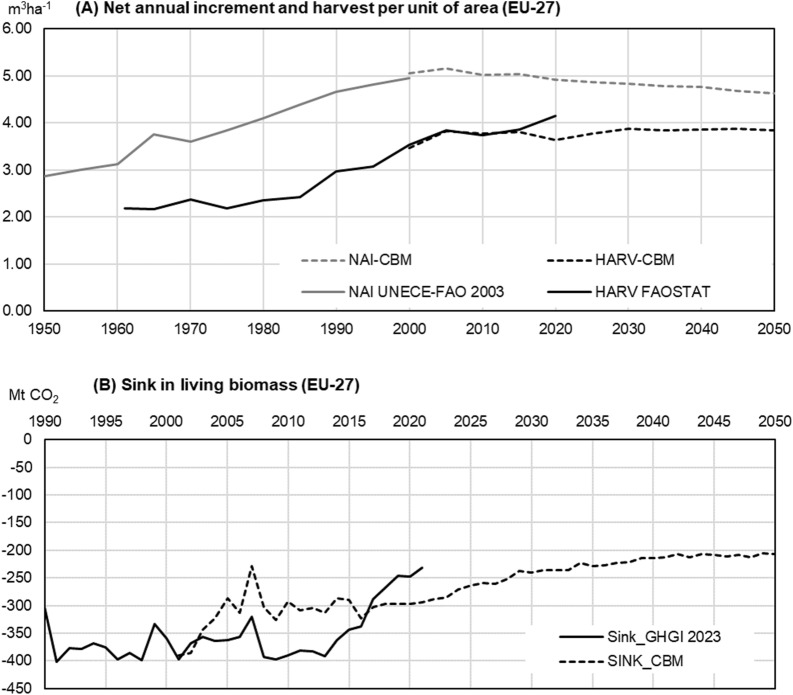
Our model’s results at the EU level, compared with reported statistics. **A** shows the net annual increment (NAI) and harvest (HARV) from the CBM modelling (see also [46] and as reported by UNECE-FAO [34] and the National Forestry Accounting Plans (NFAP) by the EU member states [9]. **B** shows the results in terms of net carbon sink in living biomass compared with the reporting of GHGI 2023 [28]

The results show EU-level harvest volume to increase steadily until 2050 (i.e., + 7% in 2050, compared with the average 2000–2015) (Fig. 6a). This development is driven especially by the forest age structure: more forests are projected to reach maturity, which leads to an increase in harvested volume if the management practices continue like those observed in the past. The increasing trend in harvests is shown for all EU sub-regions, and especially so in northern and central-western Europe, where a large forest area is reaching a mature stage. In the meantime, NAI is projected to decrease gradually (-8% in 2050, compared with the average of the period 2000–2015, Fig. 6a), because of the same ongoing ageing process of most of the European forests.

In terms of forest sink (Fig. 6b), the CBM projections correspond to a decrease of the forest sink (excluding HWP and any additional afforestation) from − 290 Mt CO_2_ in 2015 to − 240 Mt CO_2_ in 2030 (− 17% compared to 2015), and to − 207 Mt CO_2_ in 2050 (− 29% compared to 2015, see [46]). Adding HWP and new afforestation would unlikely change this trend. For comparison, the plan to plant three billion additional trees in the EU by 2030 is estimated to yield an additional sink in the order of − 15 Mt CO_2_/y by 2050 [29].

The greater decline of the forest sink in recent years reported by GHGI 2023 compared to CBM (Fig. 6b) reflects the fact that our modelling may not fully capture the forest-related country data released very recently, such as those that triggered the recent recalculations in the GHGI 2023 (Fig. 5).

It is worth noting that our modelled results cannot be directly compared to the modelling underpinning the proposal of revised LULUCF regulation [5]. First, while we focus on forest biomass, the revised LULUCF regulation includes all carbon pools and land uses. Furthermore, while we aimed to shed light to what “the continuation of past management” could lead us to, the LULUCF proposal considered what “could be feasible” with an economically optimal forest management [5]. The reference scenario modelling in [5] projects a LULUCF sink of − 258 Mt CO_2_e for 2030. Only a combination of additional mitigation, set in action by moderate carbon prices addition, results in modelled net removals of − 310 Mt CO_2_e in the LULUCF sector. Additional mitigation considered in the modelling include, for instance, increased afforestation, avoided deforestation, improved forest management, fallowing of histosoils, improved crop rotations and improved grassland management [5].

Despite the lack of direct comparability with the revised LULUCF regulation, our modelled results are relevant, because the biomass sink in existing forests has a prevalent impact on the level and trend of the whole LULUCF sink in the EU (see Fig. 1). Based on our modelling, the EU LULUCF targets for 2030 and 2050 will unlikely be met unless there are substantial and rapid changes to the current forest management practices, or the net GHG balance elsewhere in the LULUCF sector (e.g., deforestation, organic soils, wetlands, agricultural soils, etc.) improves more than assumed by the modelling that guided the setting of the targets [5].

### What can we do to improve the sinks?

The analysis of the drivers for diminishing forest sinks shows that the main culprits are decreasing increment and increasing harvest and tree mortality. To change the course, it is necessary to look at the possibilities to reverse the development of these factors, while taking into account resilience and adaptation needs [29, 47]. In addition, possible mitigation can be achieved through increasing forest area (afforestation) or avoiding deforestation.

On a forest stand level, forest increment can be enhanced through, e.g., improved plant material, tree species selection, regeneration using faster growing species, fertilization, and appropriately done thinnings [48–50]. The common challenge for enhancing forest increment is the time scale: the results are shown only in the long run over the lifetime of the forests, with only modest impacts in the timeframe of a couple decades available to respond to the climate emergency. In the meantime, climate-change driven changes to growing conditions, such as decreased precipitation, are found to have an increasingly adverse impact on forest increment [51]. The already observed changes in precipitation patterns have been observed to be particularly troublesome for spruce and other conifers sensitive to droughts [52]. Favouring mixed forests and broadleaved species over conifer monocultures are seen as preferred management choices to adapt to the changing climate, and their area is already increasing in many countries [53, 54].

Natural disturbances have already had a profound impact on the management regimes in especially central and southern Europe (see e.g. [55]). Many EU countries have faced unprecedented harvests in the recent years due to salvage logging as a response to windthrow and insect outbreaks, the latter in turn influenced by droughts. Management choices that contribute to mitigating the adverse impact of natural disturbances are therefore a key element of climate-smart forestry, where again tree species choice and mixed forest structures are considered to be most resilient against diverse threats [47, 56]. It is noteworthy that while disturbances may cause severe challenges to the economic utilization of wood, the carbon in the dead wood left on site is not released into the atmosphere immediately, but instead takes years or even decades to decay and constitutes therefore an important carbon pool. Furthermore, dead wood is currently underrepresented in most managed forests in the EU, and increasing its amount would have clear biodiversity benefits [57]. On the other hand, vast amounts of dead wood left on site, in particular after large-scale disturbances, increase the risk and severity of bark beetle outbreaks, fungi infestations and other threats to the remaining living trees. Therefore, removal of damaged trees after disturbances is typically recommended from the forest management point of view, and is in some countries also obligatory by law (see e.g. [58]).

The role of wood harvest and its recent trends in Europe is a controversial topic in the discussion on forest-related climate change mitigation (see e.g. [59–62]). Reducing harvesting is one of the rare alternatives available that has an immediate impact on the forest sinks in the short to medium term (i.e., few decades). On the other hand, harvesting and wood products support a circular bioeconomy, their use can substitute fossil emissions, sustain a greater forest growth in the future and reduce the vulnerability of forests to natural disturbances. Recent literature suggests that in the short to medium term, limiting harvests to current levels or below is a more effective climate change mitigation action than increasing harvests to produce more wood-based materials and fuel, even when considering the fossil feedstocks they substitute [63–68]. A central reason for this finding is that the current wood product portfolio is dominated by relatively short-lived products such as packaging material and paper, whose substitution effect is low compared to the decrease in forest carbon sinks due to increased harvest [65]. Furthermore, substitution effects diminish in the future with increasing decarbonisation of industries. It can be expected that in 2050 the share of fossil fuels will be rather low. Wood may thus substitute rather other renewable and recycled materials by that time, than those of fossil origin [2]. Climate change mitigation therefore calls for an industry shift towards more long-lasting wood products and improving cascading use of wood over direct energy use.

Possible options to limit the further increase of harvest levels while allowing for wood procurement for sustainable bioeconomy development include increasing rotation lengths, reducing the intensity of thinning, and paying attention to allocation and locally designed management models, including the choice of areas for nature restoration and biodiversity protection. An EU-wide analysis in [69] found that when allocated correctly, changes in the use of even-aged and selective logging, tree species changes, and set-aside areas, the forest sink in the EU could be maintained or slightly enhanced already by 2030, while maintaining the wood harvest on the level projected for the business-as-usual scenario. In southern Europe, converting management practice from coppicing to high forests is a means to store more carbon for longer time periods (see e.g. [70, 71]). Locally, even increased harvesting may be needed for adaptation purposes (e.g., replacing maladapted species), to collect wood after the natural disturbance events (salvage logging) and to reduce the amount of biomass which is at risk to be lost, e.g., during a pest outbreak or in wildfires – a risk which is materializing in Canada’s and Russia’s old-growth forests [72]. These adaptation practices need to be increasingly taken into account when planning for forest management and the potential availability of wood. The climate targets agreed at EU level focus on 2030 and 2050 also because this time period is crucial to limit future impacts of climate change and reduce the risk of breaching dangerous tipping points [73]. In this period, the sink from existing forests is particularly important to ‘buy time’ while developing a fossil-free economy. At the same time, as reflected in the EU climate policy, the contribution of forests for climate change mitigation is finite and cannot offer excuses for delayed actions in other sectors [74].

The GHG inventories act as a navigation system for measuring our location and the distance left to reach our target. If our navigation system shows a location with measurements from several years ago, it loses its usefulness, no matter of its accuracy. Recent evidence suggests that integrating Earth observation information with ground data from national forest inventories has a potential to improve the quality of the estimates and fill this temporal gap [75–77]. This would allow national GHG inventories to reflect better the recent dynamics of the forest sink, and thus to inform policy makers in a more timely manner. To this end, the expected upcoming legislation on improved forest monitoring, as mandated by the EU forest strategy [19], represents a unique opportunity to improve the accuracy and timeliness of forest-related information. Without this essential upgrade to our navigation system, tracking progress toward the EU’s climate targets becomes challenging, and realizing the central role of forests within these targets becomes increasingly difficult.

## Conclusions

Enhancing the forest sink is a likely prerequisite to meet the EU LULUCF target in 2030. This requires that the difference between the net forest increment and harvest increases over time. Our findings, based on the latest national GHG inventories and supported by our own modelling indicate, however, that the EU forest sink is clearly going in the opposite direction, therefore progressively getting off track from the path towards the LULUCF target for 2030.

In addition, the modelling of the main determinants of the forest sink—i.e. net increment and harvest – suggests that this trend will likely continue unless the current management practices rapidly change. Given the typically long response times needed to increase forest increment and the increasing risk of natural disturbances, it is of utmost importance to consider future climate change mitigation and adaptation implications in the forest management decisions that are made now. These decisions will pave the way for the central role that the land use sector, bioeconomy, and forests in particular, are expected to play in achieving climate neutrality by 2050. The enhanced ambition of the Fit-for-55 package is a step towards this direction, creating near-term targets that enable achievements of longer-term goals.

To change track rapidly, as outlined above, timely data is needed. Currently, national GHG inventories are often based on data that is collected periodically and consequently, there is a lag of several years, sometimes more than a decade, in measuring and reporting changes in the carbon sink. This lag is increasingly problematic, as it gives belated feedback both on the consequences of forest management, and on the overall strength of the sink. As highlighted by the IPCC [73], it is still possible to limit the most dangerous consequences of climate change, but we need to act rapidly through a quick phase-out of fossil emissions, and as much CO_2_ removals as possible. While it is clear that forest stocks or sinks cannot be increased indefinitely nor replace emissions reduction needs on other sectors, climate neutrality will rely on the forest sink before large-scale technical solutions for carbon capture and storage become operational.

## Methods

Our analysis aims to compare the long-term evolution of net annual increment and harvest as reported from different data sources within the historical period 1950–2015, and as estimated, through a modelling approach, from 2016 to 2050. NAI is commonly defined as the difference between the Gross Annual Increment determined within a certain period, minus annual natural losses occurred within the same interval [78]. Even if largely used, the concept of NAI is applied in several countries only for international reporting (e.g., SoEF, FAO, ESTAT) and a deep analysis of the values reported on SoEF from EU member states highlighted a lack of harmonization between various countries [51]. Despite these methodological differences, within the present study, we computed and compared the average NAI per unit of area derived from the following data sources:

1. UNECE FAO 2003: based on data reported by [34] we derived the average NAI per ha for the period 1950–2000, with 5-year time intervals, computed as the ratio between the total NAI reported in Annex 5.3 and the forest area reported in Annex 5.1. These values include all EU-27 countries, except CY, EE, LT, LV and MT and partially include some non-EU-27 country as part of former Yugoslavia.
2. SoEF 2015 [79] (for the year 2005) and SoEF 2020 [43] (for 1990, 2000, 2010 and 2015): based on the NAI reported for the Forest Area Available for Wood Supply (note that this area it is not necessarily comparable with the one considered from UNECE FAO 2003 and is different from the total forest area considered from the other data sources).
3. Ad hoc analysis of the data reported by EU member states within their National Forest Accounting Plans [9] for the reference period 2000–2009 (the average NAI is attributed to 2005) and for the compliance period 2021–2025 (the average NAI is attributed to 2022). In this last case, the estimates are based on the countries’ projections, assuming the continuation of the management practices applied within the reference period. In both cases, the NAI is estimated as:

1 $$\text{N}\text{A}\text{I}={\text{L}\text{B}}_{\text{M}\text{S}\text{C}}+\text{M}\text{R}$$

Where, LB_MSC_ is the merchantable living biomass stock change in m^3^ ha^− 1^ yr^− 1^ which can be directly derived from the net biomass C stock change (LB_SC_, in tones CO_2eq_ yr^− 1^) reported by countries’ GHGI for the historical period 2000–2009 (i.e., the so-called reference period, considered within the NFAPs) and for the Compliance Period 2021–2025, according to the following assumptions (including the average basic wood density WD and root correction factor R):

2 $${\text{LB}}_{{{\text{MSC}}}} = \left( {{\text{LB}}_{{{\text{SC}}}} {\text{*}} - \frac{{12}}{{44}}} \right){\text{*}}\frac{2}{{\overline{{{\text{WD}}}} }} + \overline{{{\text{R}}_{{{\text{CF}}^{ \to } }} }}$$

MR is the amount of merchantable biomass removed each year at country level (in m^3^ ha^− 1^ yr^− 1^) derived from the total amount of harvest (H) inferred from the data reported within NFAPs (see [9]), further corrected to exclude the share of non-merchantable wood removals (NM_wr_), as estimated from the CBM output:

3 $$\text{M}\text{R}=\left(\text{H}\text{*}{\text{N}\text{M}}_{\text{w}\text{r}}\right)$$

4. Specific estimates derived from the Carbon Budget Model (CBM), applied to the total forest area of each country (i.e. ca. 158 Mha for EU-27), deriving the evolution of NAI according to the harvest reported by FAOSTAT for the historical period 2000–2015, and for the continuation from 2016 to 2100, of the same management practices defined within the previous historical period (see [46]). This approach was used to define management rules for a Business-as-Usual scenario (BaU), resulting in an overall harvest increase by about 7% in 2050, compared to the average of the historical period. The long-term evolution of NAI (since 2016 onward) was derived, for each member state, as the net difference between the merchantable standing stock volume estimated by model on two consecutive time steps, plus the amount of removals and corresponding logging residues:

4 $$NAI = (VMt2 -VMt1) + Rt1 + LRt1$$

Where, VM_t1_ and VM_t2_ is the merchantable volume with bark estimated on two consecutive time steps, t_1_ and t_2_; Rt1 is the total amount of removals applied on time step t_1_, and LR_t1_ is the corresponding amount of logging residues. For further details on the CBM and on the overall methodological assumptions, please see [46].

In the modelling, all data were considered per unit of area, to avoid possible inconsistencies between different data sources.

## Data Availability

The data is available from the authors upon request.
